# Supplementary material for: SpecTUS: Spectral Translator for Unknown Structures Annotation from EI-MS Spectra
Source: Anal Chem. 2026 Jul 13;98(29):21670–82. doi: 10.1021/acs.analchem.6c02423 (PMC13425552; doi:10.1021/acs.analchem.6c02423)
Supplement: Supplementary file 1 [file ac6c02423_si_001.pdf]

# SpecTUS: Spectral Translator for Unknown Structures annotation from EI-MS spectra

Adam Hájek[1], Michal Starý[2], Elliott Price[3], Filip Jozefov[4], Helge Hecht[3], and Aleš Křenek[1*]

[1]Institute of Computer Science, Masaryk University, Šumavská 525/33, Brno, 602 00, Czech Republic
[2]School of Computation, Information and Technology, Technical University Munich, Boltzmannstraße 3, Garching bei München, 85748, Germany
[3]RECETOX, Masaryk University, Kamenice 753/5, Brno, 625 00, Czech Republic
[4]Faculty of Informatics, Masaryk University, Botanická 554/68a, Brno, 602 00, Czech Republic

*Email: ljocha@ics.muni.cz

## Supporting information

# A Results

## A.1 Baseline results

| | NIST | SWGDRUG | Cayman | MONA |
|---|---|---|---|---|
| $HSS_{50}$ | 45.0% | 55.2% | 58.2% | 22.2% |
| $HSS_{10}$ | 35.5% | 47.1% | 50.3% | 16.0% |
| $HSS_1$ | 18.8% | 21.0% | 23.9% | 5.9% |
| $SSS_{50}$ | 29.4% | 29.3% | 26.4% | 16.1% |
| $SSS_{10}$ | 23.2% | 23.2% | 23.5% | 11.7% |
| $SSS_1$ | 12.5% | 9.5% | 9.4% | 5.2% |

Table SI1: Percentage of cases where database search methods (SSS and HSS) successfully retrieved the closest structure from the reference database among the top-1, top-10, and top-50 suggested candidates. Performance is evaluated across all test sets: NIST test split, SWGDRUG, Cayman, and MONA.

## A.2 SpecTUS results

| | NIST | | SWGDRUG | | Cayman | | MONA | |
|---|---|---|---|---|---|---|---|---|
| | $Sim_k$ | $Acc_k$ | $Sim_k$ | $Acc_k$ | $Sim_k$ | $Acc_k$ | $Sim_k$ | $Acc_k$ |
| BDC | 0.72 | 0.0% | 0.68 | 0.0% | 0.68 | 0.0% | 0.70 | 0.0% |
| $HSS_{50}$ | 0.62 | 0.0% | 0.61 | 0.0% | 0.61 | 0.0% | 0.47 | 0.0% |
| $HSS_{10}$ | 0.57 | 0.0% | 0.58 | 0.0% | 0.57 | 0.0% | 0.42 | 0.0% |
| $HSS_1$ | 0.45 | 0.0% | 0.46 | 0.0% | 0.46 | 0.0% | 0.27 | 0.0% |
| $SSS_{50}$ | 0.55 | 0.0% | 0.50 | 0.0% | 0.47 | 0.0% | 0.44 | 0.0% |
| $SSS_{10}$ | 0.50 | 0.0% | 0.46 | 0.0% | 0.43 | 0.0% | 0.38 | 0.0% |
| $SSS_1$ | 0.39 | 0.0% | 0.34 | 0.0% | 0.34 | 0.0% | 0.26 | 0.0% |
| $SpecTUS_{50}$ | 0.84 | 69.8% | 0.86 | 64.6% | 0.78 | 45.2% | 0.58 | 37.2% |
| $SpecTUS_{10}$ | 0.81 | 65.0% | 0.82 | 58.5% | 0.74 | 38.8% | 0.54 | 34.0% |
| $SpecTUS_1$ | 0.67 | 43.3% | 0.69 | 35.0% | 0.60 | 20.7% | 0.41 | 20.8% |

Table SI2: Comparison of $Sim_k$ and $Acc_k$ metrics between the final SpecTUS model and database search methods across all testing datasets (NIST test, SWGDRUG, Cayman, MONA).

| | BDC | HSS | SSS |
|---|---|---|---|
| $SpecTUS_1$ | 46.8% / 50.8% | 76.4% / 83.6% | 79.6% / 88.1% |
| $SpecTUS_{10}$ | 69.9% / 72.2% | 84.4% / 87.3% | 87.8% / 91.2% |
| $SpecTUS_{50}$ | 75.8% / 77.9% | 85.6% / 88.0% | 88.9% / 91.9% |

Table SI3: *Win Rate / At-least-as-good Rate* of SpecTUS over database search methods on the **NIST test** set. For all values, the compared performance involves the same number of candidates, e.g., $\mathrm{ALAG}(\mathrm{SpecTUS}_{10}, \mathrm{HSS}_{10})$.

| | BDC | HSS | SSS |
|---|---|---|---|
| $\text{SpecTUS}_1$ | 44.8% / 49.5% | 72.3% / 81.2% | 87.7% / 90.2% |
| $\text{SpecTUS}_{10}$ | 70.2% / 73.7% | 81.3% / 84.9% | 88.9% / 92.3% |
| $\text{SpecTUS}_{50}$ | 77.5% / 80.7% | 85.3% / 88.0% | 90.9% / 93.1% |

Table SI4: *Win Rate* / *At-least-as-good Rate* of SpecTUS over database search methods on the **SWG-DRUG** dataset. For all values, the compared performance involves the same number of candidates, e.g., $\text{ALAG}(\text{SpecTUS}_{10}, \text{HSS}_{10})$.

| | BDC | HSS | SSS |
|---|---|---|---|
| $\text{SpecTUS}_1$ | 31.3% / 35.0% | 63.1% / 67.4% | 77.0% / 81.7% |
| $\text{SpecTUS}_{10}$ | 56.3% / 60.3% | 71.0% / 74.2% | 82.7% / 84.6% |
| $\text{SpecTUS}_{50}$ | 64.2% / 68.7% | 74.2% / 77.2% | 84.2% / 86.4% |

Table SI5: *Win Rate* / *At-least-as-good Rate* of SpecTUS over database search methods on the **Cayman** library. For all values, the compared performance involves the same number of candidates, e.g., $\text{ALAG}(\text{SpecTUS}_{10}, \text{HSS}_{10})$.

| | BDC | HSS | SSS |
|---|---|---|---|
| $\text{SpecTUS}_1$ | 21.3% / 23.4% | 65.0% / 70.6% | 65.5% / 72.4% |
| $\text{SpecTUS}_{10}$ | 35.6% / 37.4% | 63.7% / 66.8% | 66.8% / 70.4% |
| $\text{SpecTUS}_{50}$ | 39.6% / 41.6% | 61.6% / 64.9% | 64.2% / 68.0% |

Table SI6: *Win Rate* / *At-least-as-good Rate* of SpecTUS over database search methods on the **MONA** library. For all values, the compared performance involves the same number of candidates, e.g., $\text{ALAG}(\text{SpecTUS}_{10}, \text{HSS}_{10})$.

| $T$ | full | 2 | 3 | 4 | 5 | 6 | 7 | 8 | 9 | 10 |
|---|---|---|---|---|---|---|---|---|---|---|
| size | 28267 | 12340 | 7159 | 4861 | 3106 | 2170 | 1440 | 1002 | 654 | 469 |
| $\text{Sim}_{10}$ | 0.81 | 0.70 | 0.63 | 0.59 | 0.55 | 0.51 | 0.47 | 0.43 | 0.40 | 0.38 |
| $\text{Acc}_{10}$ | 65% | 44% | 33% | 27% | 21% | 17% | 13% | 9% | 6% | 4% |

Table SI7: $\text{SpecTUS}_{10}$ scenario on the **NIST** test set filtered by increasing MCES threshold $T$: reduced test set size, accuracy and similarity

# B Methods

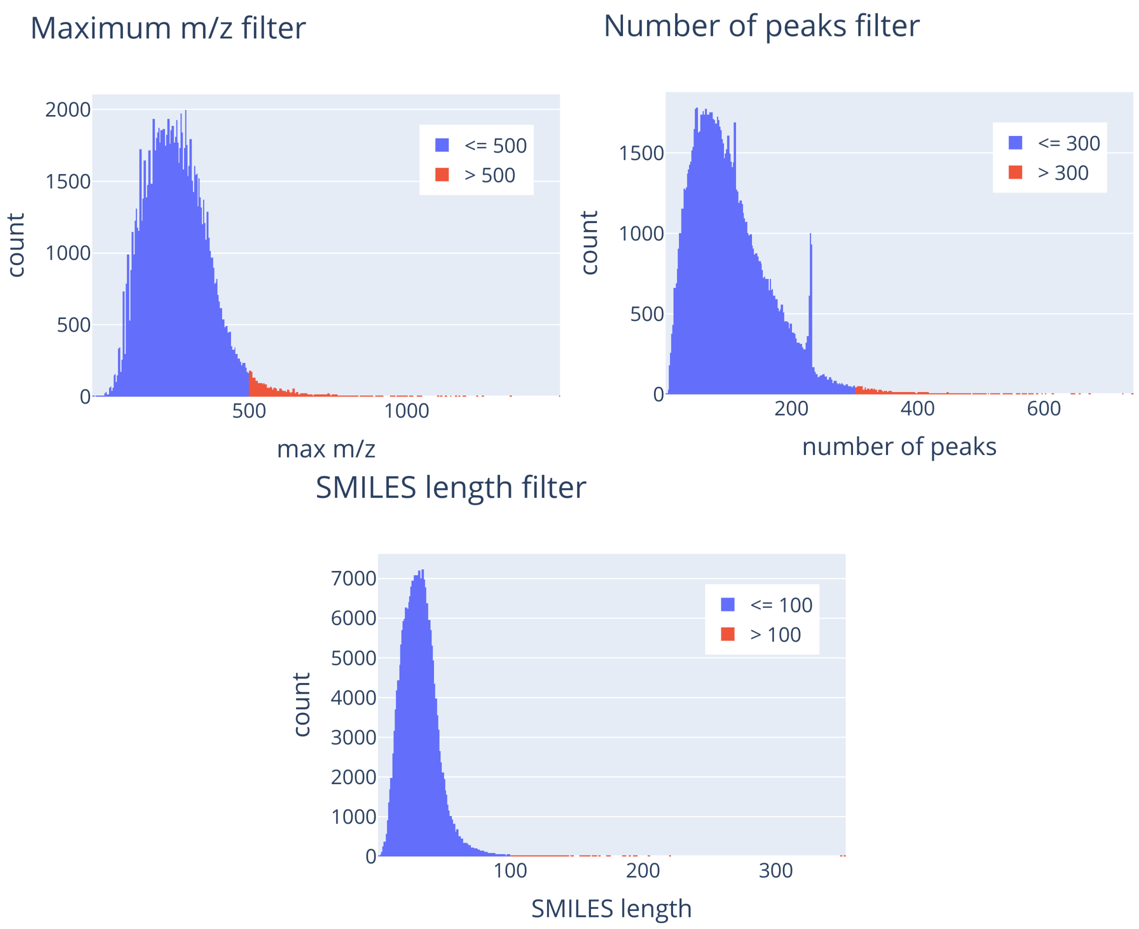


Figure SI1: Frequency plots of the three data filtering criteria, measured on the NIST train set.

# C Experiments

| | $Acc_1$ | $Acc_{10}$ | $Sim_1$ | $Sim_{10}$ |
|---|---|---|---|---|
| exp5: 8.6M_448k+296k (SpecTUS) | 43.3% | 64.8% | 0.67 | 0.81 |
| exp5: 8.6M_224k+148k | 40.7% | 62.8% | 0.65 | 0.80 |
| exp5: 4.2M_224k_148k | 39.8% | 61.8% | 0.64 | 0.79 |
| exp5: 4.2M_224k_74k | 39.1% | 61.8% | 0.64 | 0.79 |
| exp4: one src token | 38.1% | 61.4% | 0.63 | 0.79 |
| exp3: RASSP:NEIMS / exp5: 4.2M_112k_74k | 37.9% | 60.7% | 0.63 | 0.78 |
| exp3: RASSP:NEIMS:NIST | 37.6% | 60.6% | 0.62 | 0.78 |
| exp3: NEIMS-only | 36.8% | 59.7% | 0.62 | 0.77 |
| exp3: RASSP-only | 34.8% | 57.6% | 0.61 | 0.76 |
| exp1: log30bins / exp2: mf10M (char-level) / exp3: no pretraining | 28.1% | 50.7% | 0.56 | 0.72 |
| exp1: log40bins | 27.6% | 49.7% | 0.55 | 0.72 |
| exp1: lin4dec | 27.4% | 49.9% | 0.55 | 0.72 |
| exp1: lin3dec | 27.3% | 49.8% | 0.55 | 0.72 |
| exp1: log21bins | 27.2% | 49.6% | 0.55 | 0.72 |
| exp1: log10bins | 24.7% | 47.0% | 0.54 | 0.70 |
| exp2: mf10K | 24.5% | 46.4% | 0.54 | 0.70 |
| exp2: mf100 | 24.5% | 47.2% | 0.55 | 0.71 |
| exp2 mf10 | 24.0% | 46.1% | 0.54 | 0.70 |
| exp1: lin2dec | 24.0% | 46.1% | 0.53 | 0.69 |
| exp2: SELFIES | 22.3% | 41.1% | 0.49 | 0.65 |

Table SI8: Summary of all experiments conducted during the development of the final SpecTUS model. The metrics were evaluated on the NIST validation set. The experiments are ordered by $Acc_1$ values, which served as the primary criterion for assessing the results.

### C.1 Experiment 1: Intensity binning

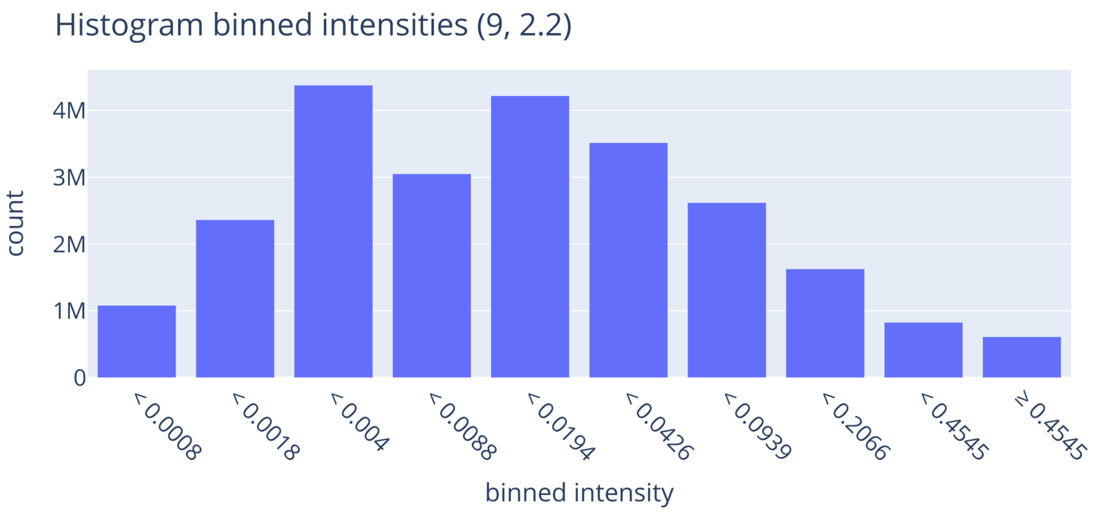


Figure SI2: Bin frequency plot after logarithmic binning with a log base of 2.2 and a shift of 9, producing 10 bins. The distribution is calculated on our train split of the NIST dataset.

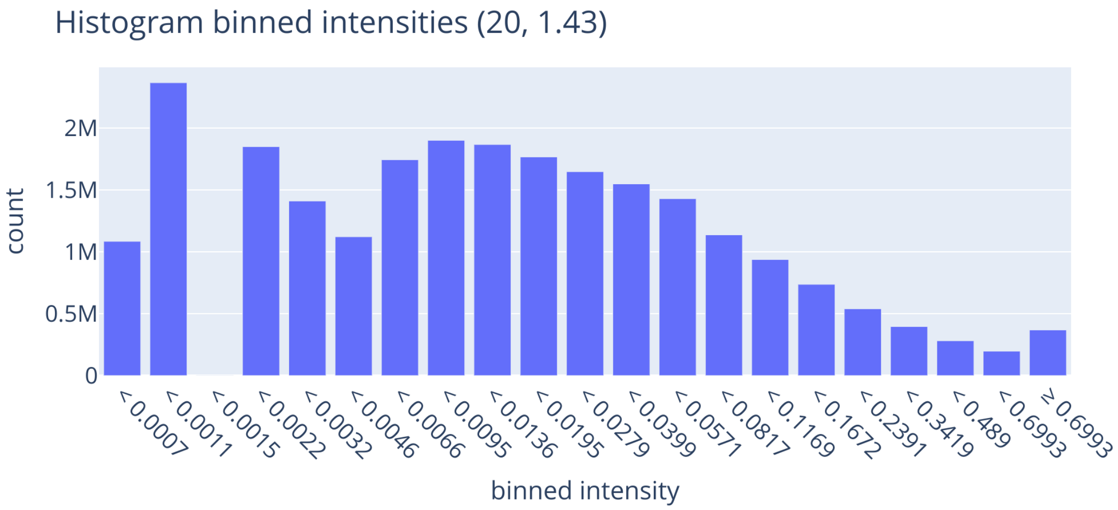


Figure SI3: Bin frequency plot after logarithmic binning with a log base of 1.43 and a shift of 20, producing 21 bins. The distribution is calculated on our train split of the NIST dataset.

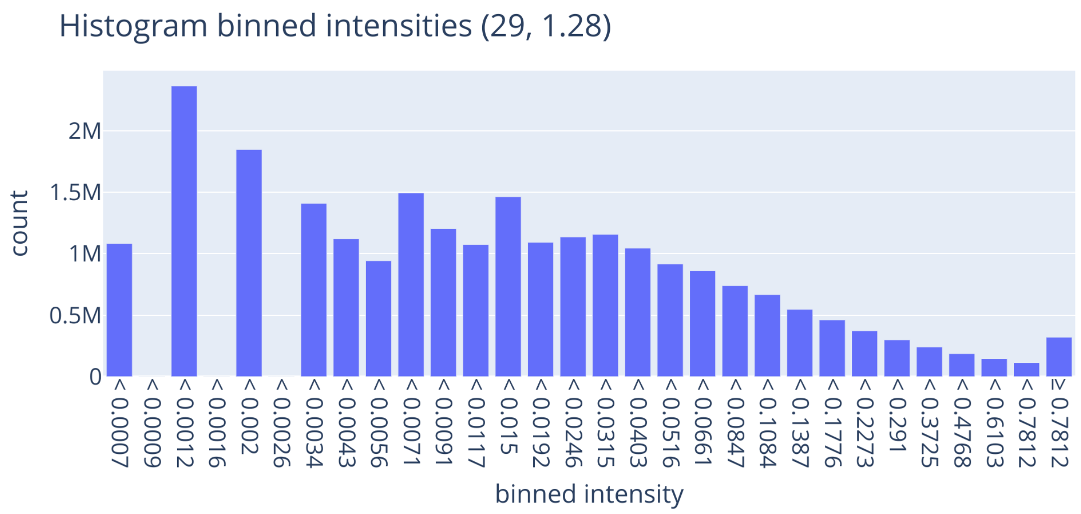


Figure SI4: Bin frequency plot after logarithmic binning with a log base of 1.28 and a shift of 29, producing 30 bins. The distribution is calculated on our train split of the NIST dataset.

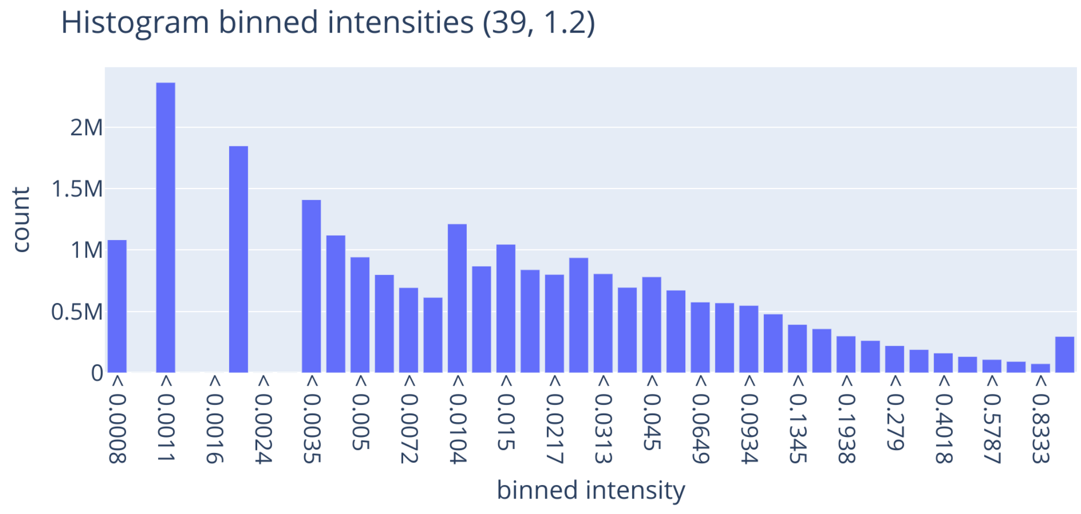


Figure SI5: Bin frequency plot after logarithmic binning with a log base of 1.2 and a shift of 39, producing 40 bins. The distribution is calculated on our train split of the NIST dataset.

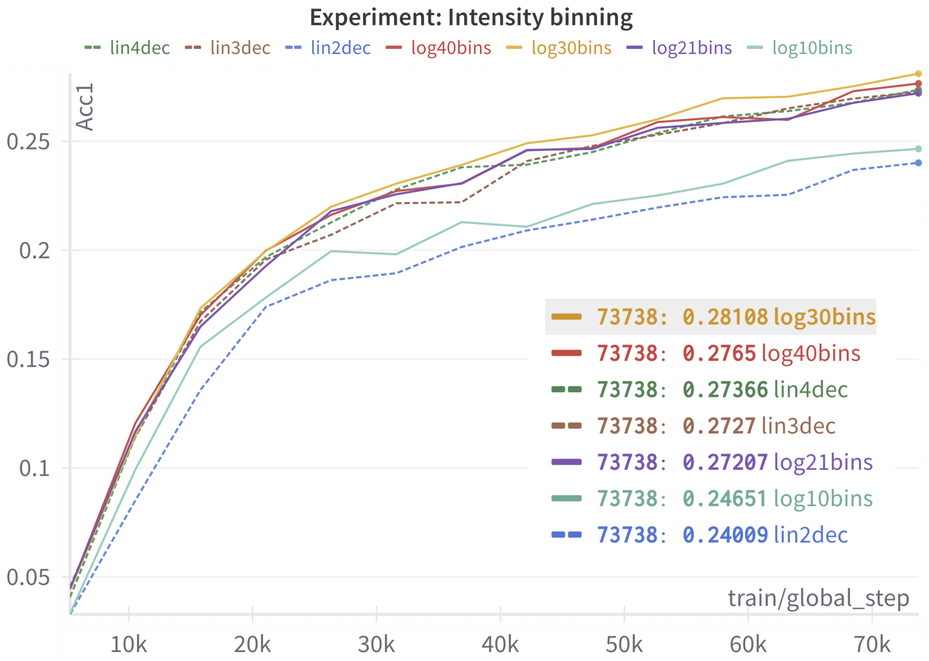


Figure SI6: Tracked control evaluations of the **intensity binning** experiment. Displays the dependency of $Acc_1$ on global step measured on the full NIST validation set. The table shows the final $Acc_1$ values.

### C.2 Experiment 2: Molecular Representations and Tokenization

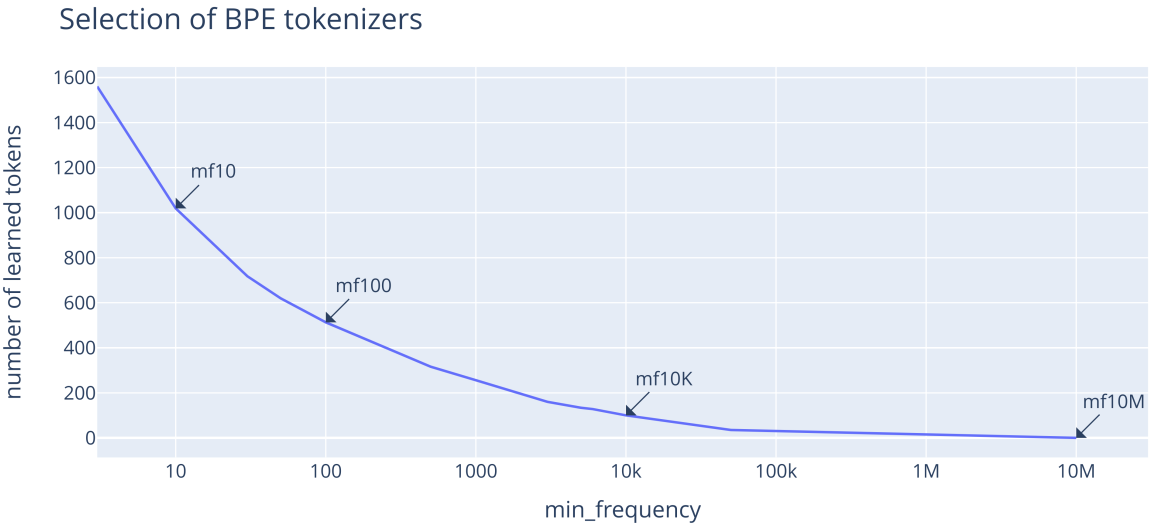


Figure SI7: Dependence of the number of learned tokens on the minimal frequency parameter. The highlighted points denote the tokenizers that we selected for examination. The x-axis is in logarithmic scale.

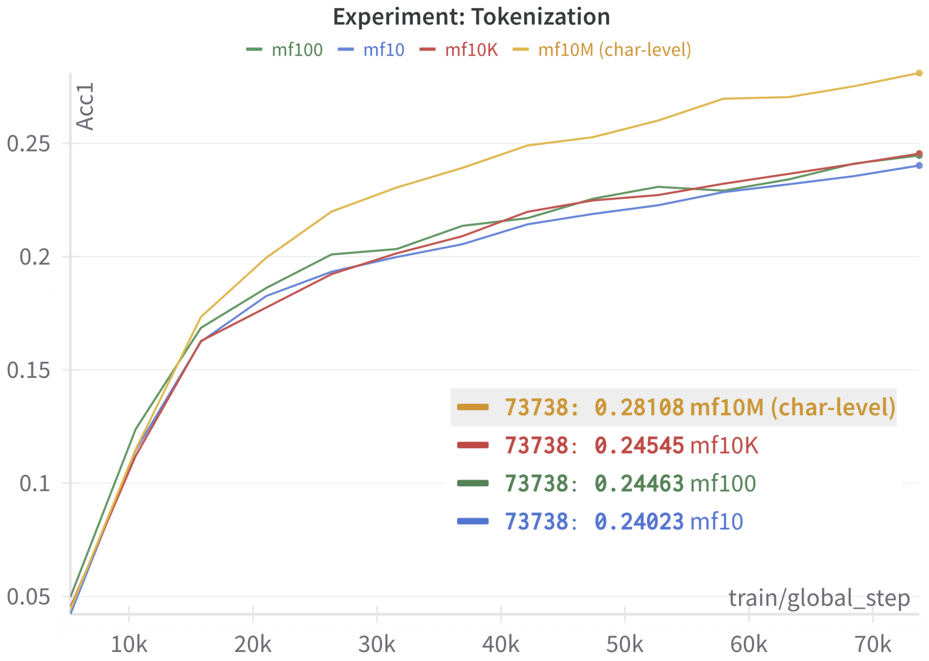


Figure SI8: Tracked control evaluations of the **tokenization** experiment. Displays the dependency of $Acc_1$ on global step measured on the full NIST validation set. The table shows the final $Acc_1$ values.

## C.3 Experiment 3: Pretraining dataset mixing

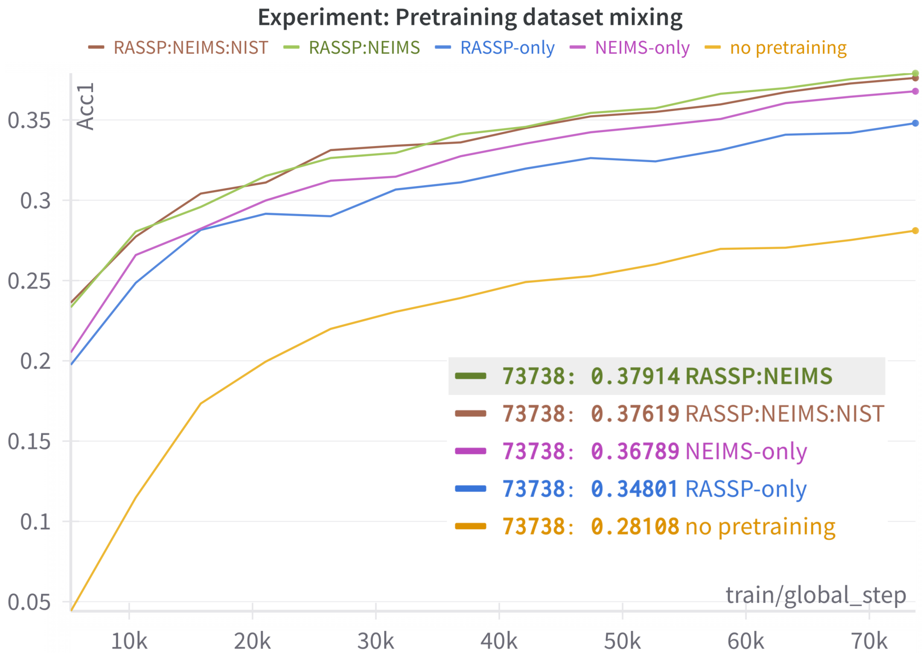


Figure SI9: Tracked control evaluations of the **pretraining dataset mixing** experiment. Displays the dependency of $Acc_1$ on global step measured on the full NIST validation set. The table shows the final $Acc_1$ values.

### C.4 Experiment 4: Source indication

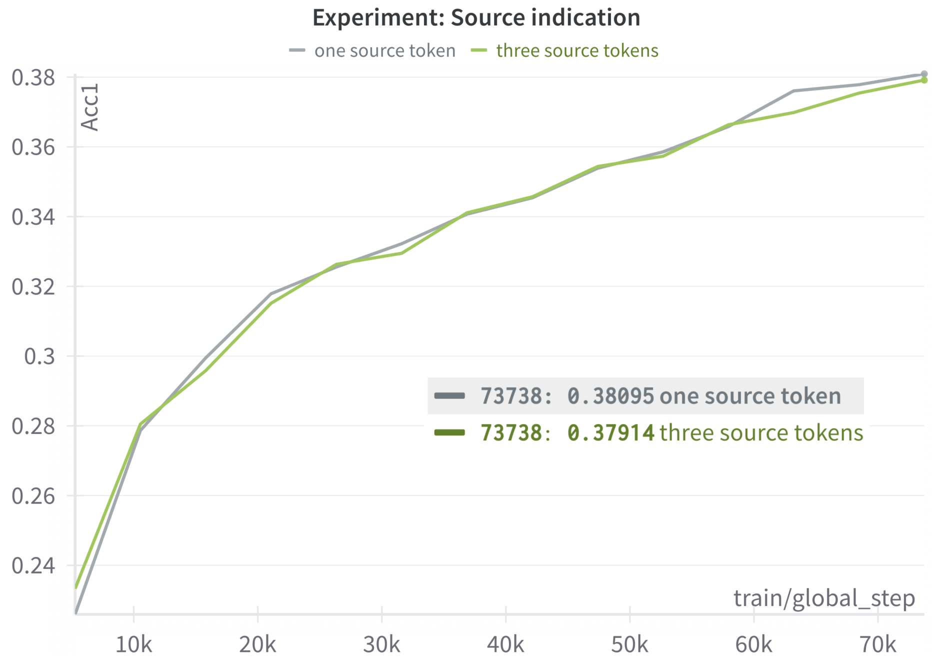


Figure SI10: Tracked control evaluations of the **source indication** experiment. Displays the dependency of $\mathrm{Acc}_1$ on global step measured on the full NIST validation set. The table shows the final $\mathrm{Acc}_1$ values.

### C.5 Experiment 5: Training length and dataset size

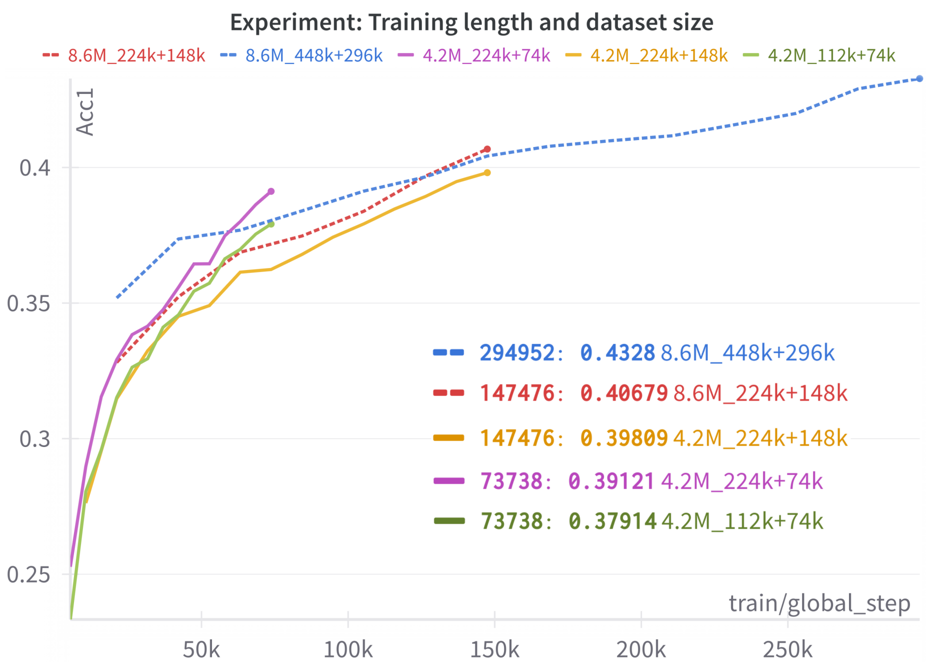


Figure SI11: Tracked control evaluations of the **training length and dataset size** experiment. Displays the dependency of $Acc_1$ on global step measured on the full NIST validation set. The table shows the final $Acc_1$ values.
